# Characterization of cortical volume and whole-brain functional connectivity in Parkinson’s disease patients: a MRI study combined with physiological aging brain changes

**DOI:** 10.3389/fnins.2024.1451948

**Published:** 2024-08-29

**Authors:** Shuaiwen Wang, Xiaoli Chen, Yanli Zhang, Yulin Gao, Lubin Gou, Junqiang Lei

**Affiliations:** ^1^Department of Radiology, The First Hospital of Lanzhou University, Lanzhou, China; ^2^Intelligent Imaging Medical Engineering Research Center of Gansu Province, Lanzhou, China; ^3^Accurate Image Collaborative Innovation International Science and Technology Cooperation Base of Gansu Province, Lanzhou, China; ^4^Gansu Province Clinical Research Center for Radiology Imaging, Lanzhou, China

**Keywords:** fMRI, Parkinson’s disease, cortex, morphology, functional connectivity

## Abstract

This study employed multiple MRI features to comprehensively evaluate the abnormalities in morphology, and functionality associated with Parkinson’s disease (PD) and distinguish them from normal physiological changes. For investigation purposes, three groups: 32 patients with PD, 42 age-matched healthy controls (HCg1), and 33 young and middle-aged controls (HCg2) were designed. The aim of the current study was to differentiate pathological cortical changes in PD from age-related physiological cortical volume changes. Integrating these findings with functional MRI changes to characterize the effects of PD on whole-brain networks. Cortical volumes in the bilateral temporal lobe, frontal lobe, and cerebellum were significantly reduced in HCg1 compared to HCg2. Although no significant differences in cortical volume were observed between PD patients and HCg1, the PD group exhibited pronounced abnormalities with significantly lower mean connectivity values compared to HCg1. Conversely, physiological functional changes in HCg1 showed markedly higher mean connectivity values than in HCg2. By integrating morphological and functional assessments, as well as network characterization of physiological aging, this study further delineates the distinct characteristics of pathological changes in PD.

## Introduction

Parkinson’s disease (PD) manifests with a diverse range of symptoms, primarily sensory-motor in nature. As the disease progresses, patients often develop a spectrum of non-motor symptoms that complicate the clinical picture. Neuropathological studies have consistently highlighted characteristic degenerative changes in the substantia nigra (SN)-striatal pathway in individuals diagnosed with PD (Furukawa et al., 2022; Pineda-Pardo et al., 2022). With advances in MRI technology, changes in the substantia nigra, such as the loss of the swallow-tail sign, are observed in susceptibility-weighted imaging (SWI) and can occur in neurodegenerative disorders such as PD, dementia with Lewy bodies (DLB) (Prasuhn et al., 2021; Tseriotis et al., 2024). Additionally, novel research methods have played an important role in studying the morphology and functional brain activity of the whole brain in PD.

In studies of brain morphology, it is well-documented that both gray and white matter atrophy are commonly observed in healthy older adults (Thambisetty et al., 2010). Numerous researchers have demonstrated that cortical volume changes due to aging are a physiological process. Understanding the distribution and timing of these morphological changes during normal aging is crucial for distinguishing between physiological and pathological alterations. Imaging studies have shown that brain volume, indicated by pixel dimensions in T1 anatomical MRI images, is significantly correlated with age-related changes in specific regions such as the dorsal prefrontal lobe, the internal olfactory cortex, and the temporal lobe (Thomann et al., 2013; Wang et al., 2019; Christova and Georgopoulos, 2023). However, studies analyzing the relationship between cortical volume and task-regulatory function have found that performance on certain tasks is not affected by normal physiological volume changes (Shaked et al., 2018). In contrast to the effects of aging on brain volume, studies of brain volume changes in people with PD have shown different volume changes in the anterior and posterior lobes of the cerebellum, areas associated with higher-level functions such as complex movement, task design, and learning (Wagner et al., 2021; van Dun et al., 2022; Kerestes et al., 2023). Some studies report no significant differences in overall brain volume between patients with the “brain-first” subtype of PD (where initial α-synuclein pathology arises inside the central nervous system) and healthy controls (Banwinkler et al., 2022). Similar findings have been reported in PD patients without cognitive impairments when compared to age-matched controls (Tessitore et al., 2012). Evidently, the theories of aging and PD brain volume changes are still at a controversial stage. Although these studies illuminate some physiological and pathological changes, the study of characteristic brain functional network changes corresponding to different morphologies might better reflect the functional characteristics represented by brain volume at different stages. This approach could enhance our understanding of the various cortical volume changes observed in patients with PD.

Functional MRI (fMRI) is a powerful tool for assessing the functional activities and interconnections between different brain regions, providing a quantitative approach for brain network research. Some studies have noted an increase in spontaneous brain region activity (ALFF) in older adults, although the connectivity between functional language networks appears to decrease (Zhang et al., 2021). As individuals age, advanced cognitive networks such as the default mode and executive control networks tend to shift from a decentralized to a more localized topology (Li et al., 2013). In contrast, the structure of primary sensory and motor networks remains relatively stable with age compared to higher cognitive networks (Thambisetty et al., 2010). Graph theory analyses by Wang et al. suggest that brain integration decreases with age, though clustering coefficients increase, preserving a stable small-world property (Alloza et al., 2018). This highlights the brain’s robustness, maintaining dynamic equilibrium through compensatory mechanisms despite regional functional declines due to aging. In PD, studies on brain functional network have not only focused on motor function symptoms but also revealed abnormalities in the brainstem-thalamus and striatal-cortical connectivity (Bostan and Strick, 2018; Johansson et al., 2022; Pimentel et al., 2023). And in PD patients with abnormal swallowtail sign, Zhou found that the substantia nigra, red nucleus, hypothalamus, thalamus, ventral striatum, caudate, lingual gyrus, postcentral gyrus, frontal pole and temporal cortex had reduced functional connectivity with voxel-mirrored homotopic correlation (VMHC) (Zhou, 2021). Additionally, enhanced functional connectivity (FC) between the cerebellum and sensorimotor network was observed in PD patients (Tolosa et al., 2021). Hyperconnectivity in motor, supplementary motor, dorsolateral prefrontal, and visual cortex has been suggested, with global efficiencies based on functional connectivity being lower in PD patients, while local efficiencies are slightly higher, potentially due to compensatory effects (Zhou, 2021). Research on non-motor symptoms has indicated significant correlations between the severity of these symptoms in PD patients and altered FC in the ipsilateral prefrontal and anterior cingulate cortex (Jellinger, 2015). Cognitive function impairments and connectivity changes in the cerebellum-sensorimotor network-dorsal attentional network have been noted as well. In PD patients with mild cognitive impairment (MCI), FC was notably reduced between the default mode network (DMN) and regions such as the middle frontal gyrus and middle temporal gyrus. Within the DMN, connections between the anterior temporal lobe and inferior frontal gyrus were also diminished (Hou et al., 2016). Further, the posterior cingulate cortex showed reduced connectivity with various regions in PD-MCI patients compared to those without cognitive impairment (Carey et al., 2021). Gorges et al. (2015) suggested that increased FC in PD patients without cognitive impairment might act as a compensatory mechanism prior to the onset of PD-MCI.

Despite the insightful findings mentioned above, they do not fully differentiate between age-related physiological intracerebral changes and those specific to PD. Consequently, this study was designed to assess and characterize cortical volume changes using voxel-based morphometric analysis (VBM). VBM is a neuroimaging technique for studying focal differences in brain anatomy, reflecting anatomical differences by quantifying the density or volume of gray and white matter for each voxel in MRI scans. To further enhance our understanding, we integrated the analysis of whole brain functional network connection using the Network-based statistic (NBS) method. It provides a comprehensive analysis of brain network integrity. Unlike methods focusing on individual region connections, NBS examines the entire brain network to identify abnormal connectivity, reflecting both physiological and pathological statuses, and has been widely utilized in studies of PD, AD, schizophrenia, and other neurological conditions (Zhan et al., 2019). By employing these approaches, we aim to comprehensively assess the pathological brain morphological features associated with physiological aging and PD, as well as the differences in whole-brain network connectivity that these features reflect.

## Methods

### Subjects

This prospective study was approved by the Ethics Committee of the First Hospital of Lanzhou University. A total of 40 patients diagnosed with PD and 83 healthy controls were recruited for the study. The controls were divided into two groups: 50 individuals aged 50–85 years, matched in age with the PD patients (HCg1), and 33 young and middle-aged adults aged 20–40 years (HCg2). PD diagnoses were confirmed by two experienced neurologists at the First Hospital of Lanzhou University, adhering to the diagnostic criteria established by the UK Parkinson’s Disease Society Brain Bank.

Exclusion criteria for participation included: (1) a history of head surgery, trauma, or neurotoxic drug use; (2) claustrophobia, depression, dementia, or history of psychotropic drug use; (3) other neurological or psychiatric disorders; (4) substantial deformation of brain structure due to cerebral infarction, hemorrhage, or atrophy that could interfere with MRI analysis; (5) excessive head movement during scanning, defined as greater than 2.5 mm movement or a rotation angle exceeding 2.5° during post-processing; (6) presence of dentures or other metallic head and facial implants. Patients with PD additionally need to be excluded those with pyramidal, cerebellar dysfunction, gaze paresis and autonomic dysfunction in neurologic examination, visual or hearing impairments, and those receiving device-assisted therapies (e.g., DBS). Ultimately, 32 PD patients and 75 healthy controls (42 in HCg1, 50–80 years old, and 33 in HCg2: 20–40 years old) were included in the study. All PD patients were assessed using the Unified Parkinson’s Disease Rating Scale, Part III (UPDRS-III), to evaluate dyskinesia and were required not to take any anti-Parkinson medication for 12 h before MRI scanning. Cognitive function was assessed using the Mini-Mental State Examination (MMSE), and handedness was evaluated with the Edinburgh Handedness Inventory. Demographic data, including age and educational background, were recorded for all participants. Each subject provided informed consent prior to their inclusion in the study.

### Image scanning

Data acquisition was conducted on a Siemens MAGNETOM Skyra 3.0 Tesla MRI scanner. The parameters setting T1-weighted structural images were acquired using the MPRAGE sequence, with parameters: TE = 2.32 ms, TR = 2,300 ms, flip angle = 8°, a 256 × 256 matrix, a slice thickness of 0.9 mm, and 192 slicers. Functional brain studies and network analyses employed BOLD sequences with echo planar imaging scans, which included settings of TE = 30 ms, TR = 3,200 ms, flip angle = 90°, matrix = 64 × 64, slice thickness = 3 mm, number of slices = 40, and 200 time points.

### Demographic and clinical data analysis

The demographic and clinical data for both the PD and HCg1 groups, HCg1 and HCg2 were analyzed using SPSS software (version 26). Categorical variables such as gender and habitual hand use were assessed using the Chi-square (Χ^2^) test, while continuous variables, including age and years of education, were analyzed using the two-sample t-test. Statistical significance was determined at a threshold of *p* < 0.01.

### Cerebral cortex volume analysis

The T1 structural images were processed using the DPABI 6.2v toolkit^1^ and SPM 12^2^ on the MATLAB 2021b platform. The images were normalized to the Montreal Neurological Institute (MNI) space in Montreal, Canada, and segmented into cortical, white matter, and cerebrospinal fluid components. For data smoothing, a full-width-at-half-maximum (FWHM) Gaussian kernel of 8 mm was applied, and the voxels were resampled to a 3 mm size. VBM was employed to assess tissue volume by quantifying the image voxels. Statistical analysis involved the use of independent samples t-tests to compare differences in cerebral cortical volume among the groups, specifically between PD and HCg1, HCg1 and HCg2. Significance levels were set at *p* < 0.05, with multiple comparison corrections applied using the false discovery rate (FDR) at *p* < 0.01. Age and years of education were included as covariates in the analysis between PD and HCg1, and years of education was included as covariates in the analysis between HCg1 and HCg2 to account for its potential influence on the results.

### Analysis of whole brain functional connection

Brain structural and functional MRI images were preprocessed and analyzed using the Brain Functional Connectivity Toolkit CONN 22a^3^ on the MATLAB 2021b platform. The preprocessing workflow in the CONN pipeline included steps such as head motion correction, unwarping, slice-timing correction, segmentation and realignment, and normalization to the MNI space. Data smoothing was achieved with an 8 mm Gaussian filter, and physiological noise was reduced using aCompCor to enhance functional connectivity results. Scans affected by excessive head motion were flagged and excluded using the artifact rejection toolbox, set at the 97th percentile.^4^ BOLD signals that deviated from the global mean by ±5 standard deviations or showed intra-frame shifts of at least 0.9 mm were considered artifacts and removed during the denoising process. In the comparison of HCg1 and HCg2, covariates in the analysis included default CONN settings, and years of education. In the comparison of PD with HCg2, covariates were added to age, in addition to the previously listed parameters. Temporal band-pass filtering was applied to isolate low-frequency fluctuations within the 0.008 to 0.09 Hz range, thereby enhancing the signal-to-noise ratio. Connectivity analysis was conducted using region-to-region connectivity (RRC) across all brain regions for comparisons between PD and HCg1, and between HCg1 and HCg2 within each brain functional network. Network-based statistics (NBS), a nonparametric, cluster-level statistical technique using graph-theoretic concepts, was employed to address multiple comparison (Zalesky et al., 2010, 2012). NBS facilitates the rejection of the null hypothesis at the network level, allowing the detection of significant network clusters rather than individual connections. In the application of NBS to examine group differences, an initial uncorrected *p*-value threshold of <0.001 was used for each contrast in connection strengths. Mean values of overall network connectivity were assessed with a threshold of *p* < 0.05 and corrected for FDR at <0.01. In this study, we analyzed whole brain networks and brain regions, including DMN, Sensorimotor Network (SMN), Visual Network (VN), Salience Network (SN), Dorsal Attention Network (DAN), Frontal Parietal Network (FPN), Language Network (LN), Dorsal Parietal Network (DPN) and the 132 brain regions and subregions (see Supplementary Table).

## Results

### Results of demographic and clinical data analysis

Statistical analysis revealed significant differences in years of education and MMSE scores between the age matched control group (HCg1) and the young and middle-aged control group (HCg2), with *p*-values less than 0.01. There was also a significant age difference between the PD group and HCg1 (*p* < 0.01). However, no significant difference in MMSE scores was observed between these two groups (see Table 1).

**Table 1:** Demographic data of the prospective study group.

	PD (*n* = 32)	HCg1 (*n* = 42)	HCg2 (*n* = 33)	*P*_PD vs. HCg1_	*P*_HCg1 vs. HCg2_
Age (years)	69.531 ± 9.456	62.261 ± 8.913	32.939 ± 5.069	0.000*	0.000*
Gender (M/F)	19/13	23/19	20/13	0.691	0.617
Education (years)	10.481 ± 3.253	10.952 ± 3.854	15.454 ± 1.519	0.842	0.000*
Dominant hand (left/right)	2/30	0/42	1/32	0.133	0.256
Disease duration (years)	5.232 ± 2.554	–	–	–	–
UPDRS-III score	23.223 ± 5.541	–	–	–	–
MMSE	27.061 ± 1.372	27.476 ± 1.200	28.182 ± 0.869	0.177	0.006*

Data are represented as Mean ± SD.

PD, Parkinson’s Disease; HC, Healthy Control; MMSE, Mini Mental State Exam; UPDRS-III, Unified Parkinson’s Disease Rating Scale Part III. *p value < 0.01.

### Analysis results for cerebral cortical volume

**Figure 1:**
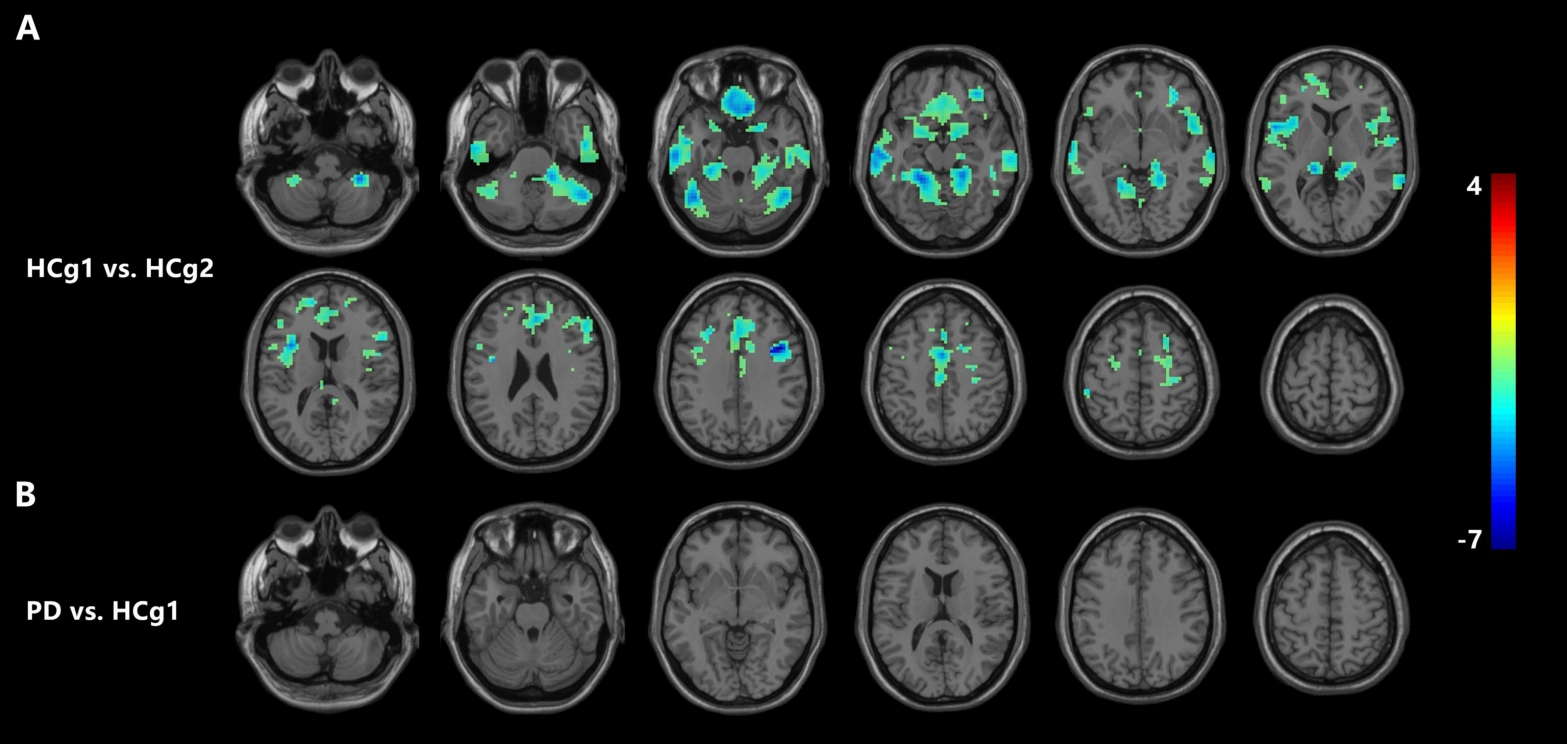
**(A)** Illustrates a significant reduction in cortical volume in HCg1 compared to HCg2 (blue area). The areas of difference include the bilateral cerebellum, frontal lobe, temporal lobe, and insula (*p* < 0.05, FDR correction *p* < 0.01). As shown in **(B)**, there was no significant change in the cortical volume of the PD group compared to the HCg1 group.

VBM analysis demonstrated that there were changes in cortical volumes between HCg1 and HCg2, notably in the bilateral cerebellum (including the anterior and posterior lobes and cerebellar regions 4 and 5, and right region 6), bilateral temporal lobes (including the middle and inferior temporal gyrus), and the medial prefrontal lobes (comprising the cingulate gyrus, superior frontal gyrus, middle frontal gyrus, inferior frontal gyrus, and parts of the motor cortex). There was also a significant reduction in the cortical volume of the bilateral insula. However, no significant changes in cortical volume were found between the PD group and HCg1 (see Table 2 and Figure 1).

**Table 2:** Analysis of cortical volume of HCg1 vs. HCg2 brains.

Brain regions		Cluster size	Coordinate	Peak value
Right cerebellum	Cerebellum anterior lobe	631	30, −45, −42	−6.659
	Cerebellum posterior lobe	456		
	Cerebelum_Crus1	272		
	Cerebelum_4_5	166		
	Cerebelum_6	127		
Left cerebellum	Cerebellum posterior lobe	259	−30, −42, −42	−6.451
	Cerebelum_Crus1	232		
	Cerebelum_4_5	174		
Left temporal lobe	Temporal_Mid	359	−63, −18, −18	−5.273
	Temporal_Inf	174		
Right temporal lobe	Temporal_Mid	311	51, −21, −27	−4.788
	Temporal_Inf	250		
Medial prefrontal lobe	Cingulum_Ant_L	151	3, 33, 30	−5.478
	Cingulum_Ant_R	62		
	Cingulum_Mid_L	141		
	Cingulum_Mid_R	136		
	Frontal_Sup_Medial_L	109		
	Frontal_Sup_R	91		
	Frontal_Sup_Orb_L	52		
	Supp_Motor_Area_L	48		
Right frontal lobe	Frontal_Inf_Oper	147	42, 9, 33	−6.761
	Frontal_Inf_Tri	128		
	Frontal_Mid	95		
	Precentral	65		
	Rolandic_Oper	65		
Right insula	Insula	68		
Left insula	Insula	141	−54, 9, 0	−5.210

L, Left; R, Right; Mid, Middle; Inf, inferior; Ant, Anterior; Sup, Superior; Orb, Orbital; Frontal_Inf_Oper, Frontal inferior opercular; Tri, Triangular; Rolandic Oper, Rolandic operculum.

### Results of functional connectivity analysis

**Figure 2:**
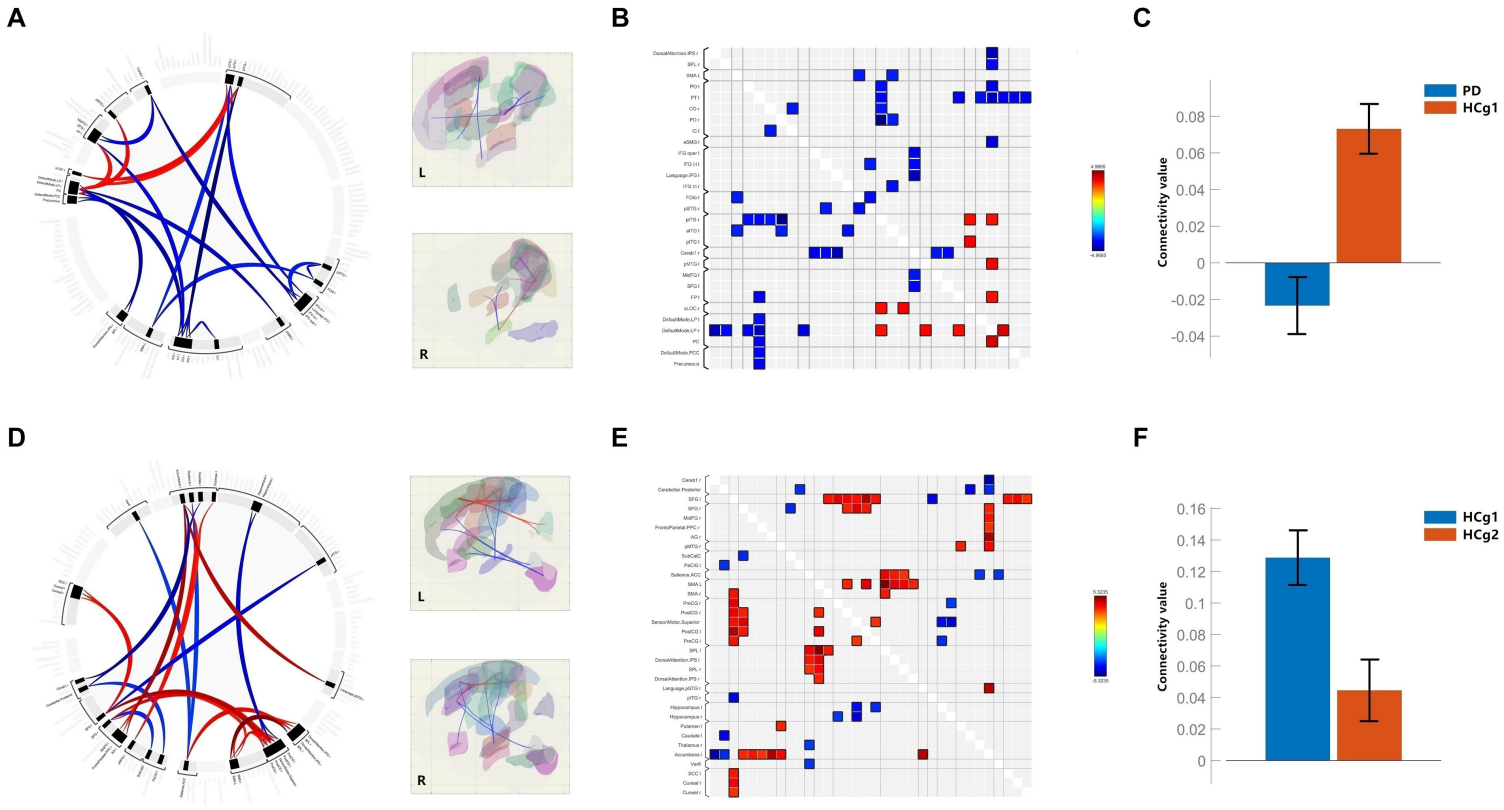
**(A–C)** Are the functional connectivity results for the comparison of PD with HCg1; **(B)** is the functional connectivity matrix plot; **(C)** is the histogram of the average functional connectivity values for both groups; **(D–F)** are the functional connectivity results for the comparison of HCg1 with HCg2; **(E)** is the functional connectivity matrix plot; F is a histogram of the mean connectivity values between HCg1 and HCg2. L/l, Left; R/r, Right; IPS=Intraparietal sulci; SPL, Superior Parietal Lobule; PO=Parietal Operculum Cortex; PT, Planum Temporale; CO=Central Opercular Cortex; IC, insular Cortex; aSMG, Supramarginal Gyrus, anterior division; IFG oper, Inferior Frontal Gyrus, pars percularis; IFG tri, Inferior Frontal Gyrus, pars triangularis; FOrb, Frontal Orbital Cortex; pSTG, Superior Temporal Gyrus, posterior division; pITG, Inferior Temporal Gyrus, posterior division; aITG, Inferior Temporal Gyrus, anterior division; Cereb1, Cerebelum Crus1; pMTG, Middle Temporal Gyrus, posterior division; MidFG, Middle Frontal Gyrus; SFG, Superior Frontal Gyrus; FP, Frontal Pole; sLOC, Lateral Occipital Cortex, superior division; LP=; PC, Cingulate Gyrus, posterior division; PCC, Posterior Cingulate Cortex; AG, Angular Gyrus; SMA, Supplementary Motor Area; PreCG, Precentral Gyrus; PosCG, Postcentral Gyrus; SPL, Superior Parietal Lobule; Ver6, Vermis 6; SCC, Supracalcarine Cortex.

In our RRC functional connectivity analysis, we observed that individuals with PD demonstrated reduced functional connectivity between the DMN, Precuneus, and various regions, including the bilateral motor cortex, parietal lobe (specifically the inferior parietal lobe within the dorsal attentional network), bilateral temporal lobe, cerebellum and frontal lobe (*p* < 0.001 uncorrected). Additionally, connectivity between the temporal and frontal lobes was notably decreased in the PD group compared to the control group (HCg1) (*p* < 0.001 uncorrected). Whereas elevated functional connectivity was seen between the parietal portion of the DMN and the bilateral temporal lobes, as well as with the left frontal pole (*p* < 0.001 uncorrected). In comparisons showing differential functional connectivity effect, the PD group showed reduced mean functional connectivity across the brain, which was statistically significant (*p* < 0.05, FDR < 0.01) (see Figure 2 and Table 3). Comparative analysis between the age matched control group (HCg1) and the young and middle-aged control group (HCg2) revealed enhanced functional connectivity in several regions. These included between the sensorimotor cortex and the left frontal lobe (including the ACC of the attentional network), between the left putamen/accumbens and the right frontal and temporal lobes, and between the visual cortex (calcarine cortex, cuneus) and the supramarginal gyrus (*p* < 0.001 uncorrected). Further, there was enhanced connectivity linking the hippocampus with the motor cortex, the attentional network with the basal ganglia, the basal ganglia with the cerebellum, the cerebellum with the paracingulate gyrus, and the subcallosal gyrus with the superior frontal gyrus (*p* < 0.001 uncorrected). Overall, the mean functional connectivity of the entire brain network was significantly higher in HCg1 compared to HCg2 (*p* < 0.05, FDR < 0.01) (see Figure 2 and Table 3).

**Table 3:** Comparative analysis of PD vs. HCg1 and HCg1 vs. HCg2 whole-brain network connectivity.

	T value	p uncorrected	p-FDR
Network_PD vs. HCg1_	−7.79	0.000	0.000
Network_HCg1 vs. HCg2_	5.37	0.000	0.000

## Discussion

In our study, we conducted a comprehensive analysis of changes in cortical volume, and brain network function due to both aging and PD. It is well-established that changes in brain volume are a physiological phenomenon throughout the aging process. This physiological state involves a complex interplay of multiple mechanisms, including protein conversion, mitochondrial metabolism, neuroinflammation, and oxidative stress, all of which contribute to the aging process (Wagner et al., 2016; Vecchio et al., 2022). When comparing the age matched control group (HCg1) with the young and middle-aged control group (HCg2), significant reductions in cortical volume were observed in HCg1 across multiple regions, including the bilateral cerebellum, temporal lobes, frontal lobes, and insula. Previous research has identified that atrophy in older adults is most pronounced in the medial prefrontal cortex (PFC), followed by the dorsolateral prefrontal cortex, temporal and parietal cortical areas, hippocampus, and caudate nucleus, which aligning with our findings (Dennis et al., 2007; Fjell and Walhovd, 2010). However, unlike neurodegenerative conditions such as AD and PD, such extensive volumetric atrophy in healthy aging has not been linked to significant cognitive and sensorimotor impairments. This suggests that physiological brain atrophy is accompanied by a dynamic equilibrium in the overall brain network, likely due to compensatory mechanisms. The network properties of the aging brain, specifically its small-world characteristics, have been the focus of several studies. These studies found that the average degree and path length differ between younger and older adults; the younger group exhibited lower average path length values, indicating a highly integrated functional network. Conversely, older adults displayed higher clustering coefficients (Goldman et al., 2012; Mancho-Fora et al., 2020), suggesting more localized connectivity. In contrast to normal aging, the brain network in PD patients demonstrates an imbalance in compensatory mechanisms, yet without significant morphological changes initially. Research has suggested that brain volume atrophy observed in later stages of PD is associated with the development of multiple complications (Lewis et al., 2016; Filippi et al., 2020). Therefore, it is critical to explore characteristic changes in brain networks in PD patients before significant volumetric changes occur, as this can provide valuable insights into the disease’s progression and associated complications.

In our continued exploration of brain function, we have focused on functional connection across various brain networks, including the DMN, SMN, VN, SN, DAN, FPN, LN, DPN and the 132 brain regions and subregions. NBS was utilized to compare the mean connectivity coefficients between groups, particularly highlighting differences between the older control group (HCg1) and the PD group, as well as between HCg1 and the young and middle-aged control group (HCg2). In patients with PD, our analysis revealed significant connectivity differences in networks, including the DMN, DAN, and LN. Functional connectivity abnormalities were predominantly noted between several brain regions within the parietal, temporal, and frontal lobes, which are primarily associated with higher-level sensory, motor, and cognitive control functions. Interestingly, these abnormal connections were not observed among the primary sensorimotor cortex and related networks such as the precentral gyrus, postcentral gyrus, and SMN. Conversely, the comparison between HCg1 and HCg2 revealed increased functional connectivity within the SMN and primary sensorimotor cortex (precentral gyrus, postcentral gyrus) and the supplementary motor area (SMA). Furthermore, the SN, LN, and DAN showed enhanced connectivity with various brain regions. These differences were more pronounced in the anterior regions of the brain, possibly correlating with the distribution of cortical atrophic areas. Additionally, the overall mean functional connectivity values were significantly higher in HCg1 compared to HCg2, suggesting an age-related compensatory mechanism in network function. Previous research has not only highlighted the prevalence of brain volume atrophy in older individuals but has also underscored the compensatory functions for cognition and language through various aspects such as neuropathology and brain networks (Lighthall et al., 2014; Di Tella et al., 2021). Furthermore, studies have noted a posterior–anterior shift in aging (PASA) that reflects a migration of active brain areas from the occipital to the frontal lobe with advancing age (Davis et al., 2008; Lamar et al., 2014; Rosjat et al., 2021). Our study also identified enhanced functional connectivity in HCg1 among multiple regions in the frontal lobe (Figures 2D–E). In the PD group, we observed negative mean network connectivity values in PD patients compared to the normal population of the same age, demonstrating a significant decline in the physiological mechanisms of brain networks. With reference to the distribution of functional brain connectivity caused by aging, most of the abnormal connectivity areas in PD patients were located in the posterior part of the brain (parietal and occipital lobes) (Figures 2A,B), suggesting a physiological imbalance in the posterior cerebral network following the onset of PD. Research has also indicated that PD patients’ brains exhibit more vulnerable small-world properties than their healthy counterparts (Suo et al., 2017; Kok et al., 2020; Zuo et al., 2023), supporting the findings of this study and highlighting the complex interplay of degenerative processes in PD.

## Limitation

A limitation of this study is the relatively small sample size and the lenient criteria for head movement during scans. Plans are underway to address these shortcomings in subsequent studies by including a larger cohort and implementing stricter movement control measures to enhance the reliability and validity of our findings. Additionally, in the analysis of brain volume in PD patients, there is a lack of exploration of cortical morphological changes in patients with later stages of the disease or in the presence of complications such as cognitive impairment. This necessitates expanding the sample size, along with follow-up and multiple MRI scans, to clarify the clinically characteristic changes in patients with PD who exhibit significant brain volume atrophy during the disease course. Combining this with functional network analysis will help us understand the specific changes in PD more clearly and assess disease progression more accurately.

## Conclusion

Through our comprehensive analysis of physiological brain morphological features, and functional networks, we have enhanced our understanding of the distinct MRI characteristics associated with PD. Notably, the volumetric analysis in this study did not reveal significant abnormalities in PD patients, but in the NBS, we observed a significant difference in the mean connectivity values of PD compared to age-matched controls (HCg1). The distribution of these connectivity discrepancy regions also differs from the distribution of network connectivity anomalies caused by aging. This may be because the cases primarily exhibit sensorimotor dysfunctions without cognitive or non-motor symptoms, resulting in less notable changes in cortical volume. However, more significant differences and distributional features have emerged in brain network functional connectivity. In future research, we aim to incorporate a study group that specifically focuses on the complexities and complications associated with PD. This approach will allow for a more thorough investigation of both morphological and functional changes linked to the disease.

## Data Availability

The original contributions presented in the study are included in the article/Supplementary material, further inquiries can be directed to the corresponding author.

## Footnotes

^1^ http://rfmri.org/dpabi

^2^ https://www.fil.ion.ucl.ac.uk/spm/software/download

^3^ https://www.nitrc.org/projects/conn

^4^ https://www.nitrc.org/projects/artifact_detect
