# Supplementary material for: Characterization of cortical volume and whole-brain functional connectivity in Parkinson’s disease patients: a MRI study combined with physiological aging brain changes
Source: Front Neurosci. 2024 Aug 29;18:1451948. doi: 10.3389/fnins.2024.1451948 (PMC11418396; doi:10.3389/fnins.2024.1451948)
Supplement: Supplementary file 1 [file Table_1.docx]

**SUPPLEMENTARY TABLE** Brain networks and brain regions used in NBS analysis

| **Brain Networks** | **Brain regions** | **Brain regions** | **Brain regions** | **Brain regions** |
| --- | --- | --- | --- | --- |
| DefaultMode.MPFC | Right Frontal Pole | Left Postcentral Gyrus | Left Lingual Gyrus | Right Hippocampus |
| DefaultMode.LP (L) | Left Frontal Pole | Right Superior Parietal Lobule | Right Temporal Fusiform Cortex, anterior division | Left Hippocampus |
| DefaultMode.LP (R) | Right Insular Cortex | Left Superior Parietal Lobule | Left Temporal Fusiform Cortex, anterior division | Right Amygdala |
| DefaultMode.PCC | Left Insular Cortex | Right Supramarginal Gyrus, anterior division | Right Temporal Fusiform Cortex, posterior division | Left Amygdala |
| SensoriMotor.Lateral (L) | Right Superior Frontal Gyrus | Left Supramarginal Gyrus, anterior division | Left Temporal Fusiform Cortex, posterior division | Right Accumbens |
| SensoriMotor.Lateral (R) | Left Superior Frontal Gyrus | Right Supramarginal Gyrus, posterior division | Right Temporal Occipital Fusiform Cortex | Left Accumbens |
| SensoriMotor.Superior | Right Middle Frontal Gyrus | Left Supramarginal Gyrus, posterior division | Left Temporal Occipital Fusiform Cortex | Brain-Stem |
| Visual.Medial | Left Middle Frontal Gyrus | Right Angular Gyrus | Right Occipital Fusiform Gyrus | Left Cerebelum Crus1 |
| Visual.Occipital | Right Inferior Frontal Gyrus, pars triangularis | Left Angular Gyrus | Left Occipital Fusiform Gyrus | Right Cerebelum Crus1 |
| Visual.Lateral (L) | Left Inferior Frontal Gyrus, pars triangularis | Right Lateral Occipital Cortex, superior division | Right Frontal Operculum Cortex | Left Cerebelum Crus2 |
| Visual.Lateral (R) | Right Inferior Frontal Gyrus, pars opercularis | Left Lateral Occipital Cortex, superior division | Left Frontal Operculum Cortex | Right Cerebelum Crus2 |
| Salience.ACC | Left Inferior Frontal Gyrus, pars opercularis | Right Lateral Occipital Cortex, inferior division | Right Central Opercular Cortex | Cerebelum 3 Left |
| Salience.AInsula (L) | Right Precentral Gyrus | Left Lateral Occipital Cortex, inferior division | Left Central Opercular Cortex | Cerebelum 3 Right |
| Salience.AInsula (R) | Left Precentral Gyrus | Right Intracalcarine Cortex | Right Parietal Operculum Cortex | Cerebelum 6 Left |
| Salience.RPFC (L) | Right Temporal Pole | Left Intracalcarine Cortex | Left Parietal Operculum Cortex | Cerebelum 6 Right |
| Salience.RPFC (R) | Left Temporal Pole | Frontal Medial Cortex | Right Planum Polare | Cerebelum 7b Left |
| Salience.SMG (L) | Right Superior Temporal Gyrus, anterior division | Right Supplementary Motor Cortex | Left Planum Polare | Cerebelum 7b Right |
| Salience.SMG (R) | Left Superior Temporal Gyrus, anterior division |  | Right Heschl's Gyrus | Cerebelum 8 Left |
| DorsalAttention.FEF (L) | Right Superior Temporal Gyrus, posterior division | Subcallosal Cortex | Left Heschl's Gyrus | Cerebelum 8 Right |
| DorsalAttention.FEF (R) | Left Superior Temporal Gyrus, posterior division | Right Paracingulate Gyrus | Right Planum Temporale | Cerebelum 9 Left |
| DorsalAttention.IPS (L) | Right Middle Temporal Gyrus, anterior division | Left Paracingulate Gyrus | Left Planum Temporale | Cerebelum 9 Right |
| DorsalAttention.IPS (R) | Left Middle Temporal Gyrus, anterior division | Cingulate Gyrus, anterior division | Right Supracalcarine Cortex | Cerebelum 10 Left |
| FrontoParietal.LPFC (L) | Right Middle Temporal Gyrus, posterior division | Cingulate Gyrus, posterior division | Left Supracalcarine Cortex | Cerebelum 10 Right |
| FrontoParietal.PPC (L) | Left Middle Temporal Gyrus, posterior division | Cortex Precuneous | Right Occipital Pole | Vermis 1 2 |
| FrontoParietal.LPFC (R) | Right Middle Temporal Gyrus, temporooccipital part | Right Cuneal Cortex | Occipital Pole Left | Vermis 3 |
| FrontoParietal.PPC (R) | Left Middle Temporal Gyrus, temporooccipital part | Left Cuneal Cortex | Right Thalamus | Vermis 4 5 |
| Language.IFG (L) | Right Inferior Temporal Gyrus, anterior division | Right Frontal Orbital Cortex | Left Thalamus | Vermis 6 |
| Language.IFG (R) | Left Inferior Temporal Gyrus, anterior division | Left Frontal Orbital Cortex | Right Caudate | Vermis 7 |
| Language.pSTG (L) | Right Inferior Temporal Gyrus, posterior division | Right Parahippocampal Gyrus, anterior division | Left Caudate | Vermis 8 |
| Language.pSTG (R) | Left Inferior Temporal Gyrus, posterior division | Left Parahippocampal Gyrus, anterior division | Right Putamen | Vermis 9 |
| Cerebellar.Anterior | Right Inferior Temporal Gyrus, temporooccipital part | Right Parahippocampal Gyrus, posterior division | Left Putamen | Vermis 10 |
| Cerebellar.Posterior | Left Inferior Temporal Gyrus, temporooccipital part | Left Parahippocampal Gyrus, posterior division | Right Pallidum |  |
|  | Right Postcentral Gyrus | Right Lingual Gyrus | Left Pallidum |  |

**MPFC:**Medial Prefrontal Cortex. **LP:**Lateral Parietal. PCC:Posterior Cingulate Cortex. **ACC:**Anterior Cingulate Cortex. **Ainsula:**Anterior insular. **RPFC:**Rostral Prefrontal Cortex **SMG:**Supramarginal Gyrus. **FEF:**Frontal Eye Field. **IPS:**Intraparietal Sulcus **LPFC:** Lateral Prefrontal Cortex. **PPC:**Posterior Parietal Cortex. **IFG:**Inferior Frontal Gyrus. **pSTG:**Superior Temporal Gyrus,posterior division. **L:**Left. **R:**Right.
